# Superior diagnostic yield of mini-laparoscopy over transjugular liver biopsy in acute-on-chronic liver failure: a case-control study

**DOI:** 10.1186/s12876-026-05401-4

**Published:** 2026-10-05

**Authors:** Louis Weisner, Malte Wehmeyer, Marcial Sebode, Julian Schulze zur Wiesch, Felix Piecha, Lennart Well, Peter Bannas, Till Clauditz, Samuel Huber, Ansgar Lohse, Peter Huebener

**Affiliations:** 1 First Department of Medicine, University Medical Center Hamburg-Eppendorf, Hamburg, Germany; 2 Department of Diagnostic and Interventional Radiology, University Medical Center Hamburg-Eppendorf, Hamburg, Germany; 3 Clinic for Diagnostic and Interventional Radiology, Albertinen Krankenhaus, Hamburg, Germany; 4 Institute of Pathology, University Medical Center Hamburg-Eppendorf, Hamburg, Germany

**Keywords:** Acute-on chronic liver failure, Liver biopsy, Pathology, Cirrhosis

## Abstract

**Background & Aims:**

Hepatic failure frequently requires liver biopsy for etiologic classification and delimitation from differential diagnoses to inform treatment decisions. Both mini-laparoscopic (MB) and transjugular liver biopsy (TB) are considered safe diagnostic modalities in liver cirrhosis (LC) patients but may associate with varying success and complication rates. We aimed to characterize the clinical values of MB and TB in patients with LC and particularly acute-on chronic liver failure (ACLF), where clinical acuity and patient risk are high.

**Methods:**

In this retrospective study, we analyzed 73 consecutive adult ACLF patients undergoing liver biopsy (57x ACLF-MB, 16x ACLF-TB) and performed 1:2-matching with non-ACLF LC patients for either biopsy modality to assess technical success, complications, and diagnostic versatility.

**Results:**

Baseline disease severity was comparable between biopsy modalities (MELD score: ACLF-MB 27 [22–33] vs. ACLF-TB 27 [19.75–32], p = 0.640). MB and TB were successfully completed in the large majority (MB 169/171 vs. TB 48/48; p = n.s.) of procedures. While the presence of ACLF associated with a higher rate of minor complications following MB (ACLF-MB: 80.7% vs. LC-MB: 52.6%, p < 0.001), major biopsy-related complications were generally rare, and their occurrence did not differ between biopsy modalities. MB-specimens were technically evaluable in 100% of successfully performed procedures, contrasting 31.3% of technical failures in ACLF-TB and 37.5% in LC-TB (all p < 0.001), respectively. In suitable biopsies, failure to obtain a histological diagnosis was observed in 3.6% of ACLF-MB, 6.2% of ACLF-TB, 17.7% of LC-MB, and 6.2% of LC-TB cases, ultimately resulting in higher rates of established or corrected diagnoses via MB than TB.

**Conclusions:**

MB provides higher diagnostic yield than TB at the cost of increased minor complication rates in ACLF. Where locally available and technically feasible, MB may be considered the preferred route of liver biopsy in end-stage chronic liver disease.

**Supplementary Information:**

The online version contains supplementary material available at https://doi.org/10.1186/s12876-026-05401-4.

## Introduction

Acute-on-chronic liver failure (ACLF) is a frequent and severe complication of end-stage liver disease and associates with excessively high short-term mortality rates [1]. It is frequently triggered by extrahepatic insults, including infections or gastrointestinal hemorrhage, but may also be precipitated by direct injury to the liver, including infectious, immunological, malignant or metabolic diseases, and no trigger can be identified in a staggering ~ 40% of ACLF patients [1, 2]. Importantly, the clinical presentation of ACLF may resemble fulminant hepatic failure (FHF), which needs careful diagnostic delineation from ACLF due to important therapeutic and prognostic implications including allocative prioritization for liver transplantation [3, 4].

In patients with hepatic failure and ambiguous non-invasive testing, the decision to proceed to liver biopsy is often impeded by an apprehension of periprocedural complications [5]. As such, liver biopsy is frequently [6, 7] - albeit not uniformly [8] - categorized as a high-risk procedure in cirrhotic patients. Particularly bleeding risk estimates exhibit significant variability, in part attributable to its marked dependency on clinical co-variables: whereas in stable cirrhosis, percutaneous liver biopsy has been associated with a bleeding risk of 0.6%-5% [9, 10, 11, 5], postprocedural bleeding complications are substantially more frequent in patients with ACLF, particularly in the presence of severe portal hypertension, infection and/or kidney failure [12, 13]. Paucity of clinical data from ACLF patients results in uncertainty regarding a suitable periprocedural management of coagulopathy in this patient subgroup [14, 15]. Consequently, transjugular liver biopsy (TB) is frequently preferred in patients with decompensated cirrhosis, particularly in the presence of ascites and coagulopathy [16], but has been associated with a lower number of assessable portal tracts [17]. Fragmentation of the obtained tissue remains an additional technical concern in TB and may be aggravated in the setting of advanced fibrosis and necroinflammation to further reduce informational value, particularly in ACLF livers [18]. The decision whether and how to proceed to liver biopsy thus also needs to weigh in variable diagnostic value of tissue samples, depending on biopsy modality and size, but also the extent of underlying liver pathology [19, 20]. Mini-laparoscopy guided liver biopsy (MB) requires significant technical and personal infrastructure, impeding its widespread implementation into clinical practice, but has been attributed with superior safety and quality profiles in patients with advanced stable liver disease [21, 22]. Here, we aimed to outline the role of TJ and MB in the management of ACLF patients, by primarily addressing issues of patient safety and diagnostic yield, to better understand the benefits and limitations of both diagnostic modalities in a context of high clinical acuity, advanced tissue degeneration and significant patient risk.

## Patients and methods

### Patient cohort

In this retrospective, single-center observational study, we included all adult (age ≥ 18 years) patients with EASL CLIF-defined ACLF [1] undergoing MB or TB at the University Medical Center Hamburg-Eppendorf (UKE) between 01/2012 and 12/2020. Liver cirrhosis was diagnosed histologically or based on conclusive medical history, imaging, laboratory studies and/or liver elastography. We identified *n* = 57 ACLF patients with ACLF-MB, and *n* = 16 with ACLF-TB. We then performed a 1:2 matched pair-analysis with liver cirrhosis (LC) patients without ACLF undergoing the same respective biopsy modalities at our institution during the same time period. Matching criteria were patient age and gender, resulting in *n* = 114 LC-MB and *n* = 32 LC-TB patients included in the analysis. All data were obtained from electronic medical records including external documentation regarding patient history and underlying medical conditions. With regards to the actual biopsies, perioperative use of coagulation factors, number of liver biopsies and obtained samples were recorded. In terms of periprocedural complications, we analyzed use of blood products and coagulation factors after the procedures for up to 7 days, as well as evidence for clinical deterioration including liver disease scores, admission to an intensive care unit (ICU), and radiological or surgical interventions in the observation period. Pathology reports were reviewed for diagnostic evaluability and suitability to obtain a definite histological diagnosis. All analyses were performed in accordance with the Declaration of Helsinki and relevant guidelines and regulations. The study was submitted to the Medical Ethics Committee of the Hamburg Medical Association (Ärztekammer Hamburg, Weidestrasse 122b, 22083 Hamburg, Germany; reference no. 2025-300598-WF). Owing to the retrospective analysis of anonymized data, the committee determined that formal ethics approval and individual informed consent to participate were not required.

### Biopsy procedures

For MB, the abdominal wall was punctured with a Veress needle, and pneumoperitoneum was established with nitrous oxide. Liver biopsy was performed under visual control with a 16G (10.6%) or a 18G needle (89.4% of procedures), respectively. Compression of the puncture site or argon plasma coagulation (Apc) were used at the interventional physician´s discretion to control bleeding, if needed, followed by deflation of pneumoperitoneum. For TB, usually the right internal jugular vein was punctured using an 18 G needle. A 0.035-inch guidewire was then advanced through the right atrium under fluoroscopic guidance, and a 9 F–7 F sheath was subsequently inserted. A diagnostic catheter was advanced under fluoroscopic control into the middle or right hepatic vein. The Ross biopsy needle was then inserted over a stiff exchange guidewire (0.035 or 0.038 inch). In 93.8% of procedures a Ross biopsy needle (15 Gauge, 9 F Sheath) was used. If biopsy was unsuccessful, an 18 Gauge needle (7 F Sheath) was used for a subsequent attempt (6.2% of procedures).

### Biopsy quality

Biopsy quality was routinely evaluated as part of the pathology report in terms of two categories: (i) *Technical sample quality* refers to the suitability of a specimen for histopathological analysis, and is mainly affected by tissue fragmentation, number of observable portal fields, and presence of blood clots. Thus, *technical failure* is defined as inability of histopathological evaluation - judged by the examining pathologist - for insufficient technical quality. Conversely, (ii) *interpretability* describes the derivation of a histopathological diagnosis from a technically adequate sample and can be restricted by extensive inflammation or advanced fibrosis obscuring underlying histopathological patterns. *Diagnostic failure* was thus defined as the failure to establish a diagnosis despite a technically adequate tissue specimen.

### Complications

Intraprocedural bleeding was categorized as minor or major bleeding according to the assessment of the performing gastroenterologist. Bleedings of minor severity were self-limiting, whereas major bleedings required interventional hemostasis, usually via APC therapy. Intra-procedural bleeding with successful hemostasis during mini-laparoscopy was not regarded as a complication. Post-procedural complications were classified according to the Clavien-Dindo grade of surgical complications [23]: Grade I - minor secondary complications, i.e. hypotension unelated to bleeding, administration of pain killers, hematoma of the abdominal wall and/or puncture site, bleeding puncture site, and nausea or vomiting, Grade II - requirement of transfusion during first week after intervention, Grade III - need for surgical, endoscopic or radiological interventions, Grade IV – ICU admission and Grade V – procedure-related death. Furthermore, grades were summarized into minor (grades I or II) and major (grades III-V) complications.

### Statistical analysis

Statistical Analysis was performed by IBM SPSS Statistics version 29.0.1.1. For analysis of continuous variables, a t-test was used, while categorial variables were analyzed by Pearson-x^2^-test or Fisher´s exact test. Continuous clinical variables were non-normally distributed (Shapiro–Wilk * p<0.05*) and are presented as median [25th–75th percentile]; between-group comparisons used the Mann–Whitney U test. Analyses of MELD-score trajectories were restricted to patients with measurements at baseline and 72 h. As within-patient MELD-score changes were non-normally distributed, changes over time were assessed within groups using the Wilcoxon signed-rank test; MELD scores are reported as median [25th–75th percentile] at each time point.

Peri-interventional blood-product and coagulation-factor administration was analyzed using a two-part approach because of substantial zero inflation. Receipt of each product was reported as n (%) and compared between groups using two-sided Fisher’s exact tests. Among recipients, administered amounts were summarized as median [25th–75th percentile]. Given the small number of recipients for several products, these quantities were assessed descriptively without inferential between-group comparisons. P-values < 0.05 were considered statistically significant.

Finally, a binary logistic regression analysis was performed to identify independent predictors of relevant postinterventional bleeding, defined as any bleeding event resulting in transfusion of packed red blood cells, admission to the intensive care unit (both within 7 days after intervention), or death (within 28 days after intervention). Variables showing an association with the outcome at a significance level of *p* < 0.20 in univariate analysis were included in a multivariate model. In addition to variables with a univariate p-value < 0.20, presence of portal hypertension (*p* = 0.3555, evidenced by splenomegaly, ascites, retrograde portal vein flow and/or presence of esophageal varices etc.) was included due to its well-documented role as a risk factor of postprocedural bleeding. Finally, the type of biopsy (*p* = 0.448) was included in the final analysis, since the comparison between ML and TB constituted a primary objective of this study. Model fit was assessed using the Hosmer–Lemeshow goodness-of-fit test, and multicollinearity was ruled out based on tolerance (> 0.1) and variance inflation factor (VIF < 5). Results are presented as odds ratios (OR) with 95% confidence intervals (CI).

## Results

### Patient characteristics

Patient characteristics are outlined in Table 1. Within the ACLF cohort, baseline characteristics were broadly comparable between biopsy modalities, with no statistically significant differences in MELD score, INR, serum bilirubin, platelet count, creatinine, CLIF-C ACLF score, or Charlson Comorbidity Index between ACLF-MB and ACLF-TB patients. In the non-ACLF LC cohort, LC-MB patients were significantly older than LC-TB patients, while LC-TB patients had a significantly higher BMI. LC-MB patients also had higher CLIF-C AD scores, whereas MELD score and other laboratory parameters did not differ significantly between biopsy modalities. Average BMI was higher in the LC-TB group, where severe obesity constituted a frequent rationale for preferring TB over MB. Etiologic clarification of underlying liver disease constituted the most prevalent indication for biopsy, followed by verification of cirrhosis or suspected malignancy (Supplementary Table ST1). In brief, biopsy was most frequently indicated for a previously unclear underlying etiology (57.9–84.4% of patients across groups), followed by suspected alcohol-related liver disease as the most common specific clinical suspicion in the ACLF cohorts (28.1% ACLF-MB, 31.3% ACLF-TB) and in LC-MB (9.6%); previously unknown, incidentally suspected cirrhosis prompted biopsy in 5.3% of ACLF-MB, 18.8% of ACLF-TB, 33.3% of LC-MB and 21.9% of LC-TB patients.

**Table 1:** Patient characteristics

Characteristic	ACLF-MB (*n* = 57)	ACLF-TB (*n* = 16)	*p* value (ACLF-MB vs. ACLF-TB)	LC-MB (*n* = 114)	LC-TB (*n* = 32)	*p* value (LC-MB vs. LC-TB)
Age, years	56 [49–61.5]	60 [54–63]	0.214	57 [48–65.25]	53.5 [39.75–61.5]	**0.023**
BMI, kg/m²	25.95 [22.47–30.91]	26.55 [25.37–28.85]	0.785	25.06 [22.73–28.41]	29.54 [25.20–40.64]	**< 0.001**
Platelet count, ×10⁹/L	115 [68.5–166.5]	108 [87–212.25]	0.749	121 [87.5–187]	96 [58.75–166]	0.059
INR	1.65 [1.37–2.27]	1.45 [1.20–2.10]	0.076	1.20 [1.09–1.47]	1.23 [1.10–1.36]	0.932
Creatinine, mg/dL	1.80 [1.19–3.00]	2.21 [1.60–2.98]	0.277	0.83 [0.70–1.10]	0.86 [0.66–1.20]	0.991
Bilirubin, mg/dL	15.65 [7.08–24.08]	12.90 [1.10–22.80]	0.167	1.45 [0.80–4.25]	1.25 [0.625–2.45]	0.097
MELD score	27 [22–33]	27 [19.75–32]	0.640	11 [8–17]	11 [8–14]	0.463
CLIF-C ACLF score	47 [44–51]	51.5 [41.25–58.25]	0.603	–	–	–
CLIF-C AD score	–	–	–	48 [42–53]	43 [38.5–45]	**< 0.001**
Charlson Comorbidity Index	4 [3–6]	5 [4–6]	0.221	4 [3–6]	4 [3–6]	0.933

Table legend: Values are presented as median [interquartile range, IQR]. P values for continuous variables were calculated using two-sided Mann–Whitney U tests. MB = mini-laparoscopic liver biopsy; TB = transjugular liver biopsy; ACLF = acute-on-chronic liver failure; LC = liver cirrhosis; MELD = Model for End-Stage Liver Disease; CLIF-C = Chronic Liver Failure Consortium; AD = acute decompensation; INR = international normalized ratio

### Patient cohort

Of *n* = 73 ACLF patients undergoing liver biopsy, *n* = 16 underwent ACLF-TB, and *n* = 57 underwent ACLF-MB. A large fraction of TB was performed in conjunction with wedged hepatic pressure gradient measurements (19/48 = 39.6%), during implantation of a transjugular intrahepatic portosystemic shunt (TIPS, 8/48 = 16.7%), or during TIPS recanalization or dilation (2/48 = 4.2%). In additional 8/48 cases, TB was indicated due to morbid obesity of patients technically precluding MB. Other indications for TB included a patient history of abdominal surgery (4/48 = 8.3%), hematologic malignancies and related bone marrow disorders (combined 4/48 = 8.3%), or therapeutic anticoagulation (1/48 = 2.1%), among others.

### Biopsy quality

In total, 80 individual liver biopsies were obtained during 57 ACLF-MB procedures, with a predominance of left liver lobe (53x) over the right liver lobe (27x). One ACLF-MB (1/57 = 1.7%) was technically unsuccessful due to postoperative abdominal adhesions precluding visualization of the liver, whereas all (16/16) ACLF-TB were successfully completed (*p* = 1.000). During 16 ACLF-TB procedures, 24 individual liver biopsies were obtained, predominantly (*n* = 20) from the right liver lobe. An average of 1.6 ± 1.0 samples were obtained during ACLF-MB versus 3.7 ± 2.7 samples during ACLF-TB (*p* < 0.001). Average core-lengths of the obtained biopsy specimens were 1.6 ± 0.4 cm (ACLF-MB) vs. 0.6 ± 0.4 cm (ACLF-TB) (*p* < 0.001). All (56/56) samples obtained via ACLF-MB were deemed technically successful. In contrast, 5 out of 16 samples (31.3%) obtained via ACLF-TB were judged technical failures (*p* < 0.001), primarily due to severe fragmentation (4/5) or the widespread presence of blood clots (1/5). In the ACLF-MB group, diagnostic failure occurred in 2/56 cases (3.6%) due to advanced fibrosis or extensive inflammatory changes, compared to 1/16 cases (6.2%) in the ACLF-TB group (*p* = 0.535). Consequently, the underlying etiology could not be determined in 3/57 ACLF-MB patients (including one laparoscopy without biopsy, cf. above: 5.3%), compared to 6/16 ACLF-TB patients (37.5%) (*p* = 0.003). In a complementary sensitivity analysis restricted to the subset of patients in whom biopsy was specifically indicated because the underlying etiology was unclear at baseline (Supplementary Table ST2), MB established a diagnosis significantly more often than TB in the ACLF cohort (28/32 [87.5%] vs. 4/8 [50.0%]; *p* = 0.037). Among patients in whom biopsy was indicated for previously unknown/suspected cirrhosis, both modalities established the diagnosis in all ACLF cases (3/3 each).

Similarly, among patients with non-ACLF liver cirrhosis (LC), one LC-MB procedure (1/114, 0.9%) had to be aborted due to large volume ascites, whereas all transjugular biopsies (LC-TB, 32/32) were successfully completed (*p* = 1.000). In the 114 LC-MB procedures, 181 liver biopsies were retrieved, predominantly from the left lobe (108 vs. 72 from the right lobe, 1x not specified). In comparison, during 32 LC-TB procedures, 34 liver biopsies were performed, predominantly (*n* = 28) from the right lobe. TB specimens were significantly more fragmented, resulting in a higher number of fragments per patient (LC-TB: 5.0 ± 3.0 vs. LC-MB: 1.7 ± 0.8; *p* < 0.001), while the average core length was significantly greater in LC-MB samples (1.6 ± 0.4 cm vs. 0.6 ± 0.5 cm for LC-TB; *p* < 0.001). All samples obtained from LC-MB (113/113) were judged technically successful and suitable for histopathological evaluation, whereas technical failure occurred in 12/32 (37.5%) of LC-TB samples (*p* < 0.001), predominantly due to severe fragmentation (11/12) or the presence of blood clots (1/12). Diagnostic failure occurred in 20/113 (17.7%) of LC-MB cases due to extensive fibrosis or inflammatory changes, compared to 2/32 (6.2%) in the LC-TB group (*p* = 0.111). The underlying etiology could thus not be determined in 14/32 (43.8%) of LC-TB patients versus 21/114 (including one laparoscopy without biopsy, see above: 18.4%) of LC-MB patients (*p* = 0.005). A concordant, non-significant trend was observed in the LC cohort when restricting analysis to patients with baseline-unclear etiology (49/69 [71.0%] MB vs. 14/27 [51.9%] TB; *p* = 0.076; Supplementary Table ST2); among LC patients in whom biopsy was indicated for previously unknown/suspected cirrhosis, MB numerically outperformed TB (34/38 [89.5%] vs. 5/7 [71.4%]; *p* = 0.230). Beyond diagnostic yield, liver biopsy also carried discernible therapeutic consequences across groups (Supplementary Table ST3): biopsy findings prompted liver-transplantation evaluation or planning in 17.9% (10/56) of ACLF-MB and 6.3% (1/16) of ACLF-TB patients (*p* = 0.436), and in 11.4% (13/114) of LC-MB and 3.1% (1/32) of LC-TB patients (*p* = 0.305); treatment was discontinued or adjusted based on biopsy findings in 8.9% (5/56) of ACLF-MB and 12.5% (2/16) of ACLF-TB patients (*p* = 0.647), and in 5.3% (6/114) of LC-MB and 0.0% (0/32) of LC-TB patients (*p* = 0.339); and biopsy contributed to excluding or detecting malignancy in 5.4% (3/56) of ACLF-MB and 5.3% (6/114) of LC-MB patients, with no such cases observed among ACLF-TB or LC-TB patients in our cohort. None of these between-modality differences reached statistical significance, likely reflecting limited statistical power given the relatively low event rates and small TB subgroups.

### Periprocedural proceedings and postinterventional complications

Transfusion of blood products and/or coagulation factors within 72 h prior to the respective biopsies are summarized and compared in Supplementary Table ST4**.** Pre-interventional administration was more frequent in ACLF than in LC patients for several products, whereas differences between biopsy modalities within the ACLF and LC cohorts were less pronounced.

**Fig. 1:**
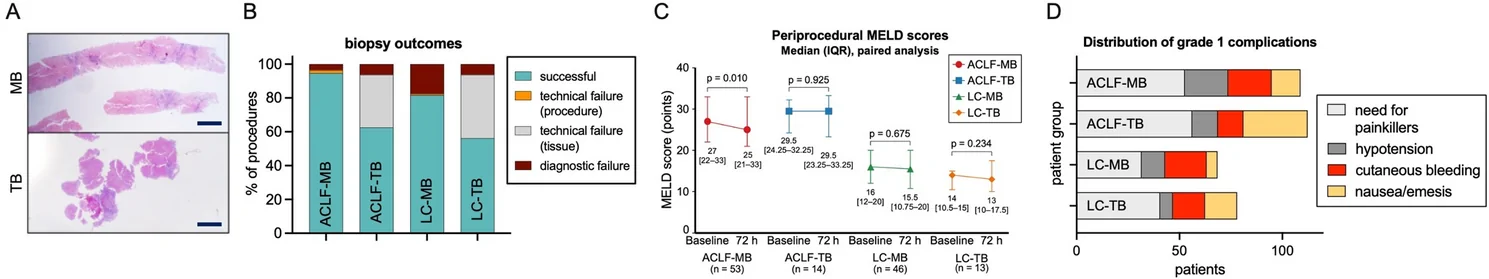
Overview of biopsy results. **A** representative photomicrographs of liver biopsy specimens obtained from mini-laparoscopic (MB) and transjugular (TB) liver biopsies. Bar = 1.000 μm. **B** Results from biopsy procedures in the respective patient/biopsy groups, shown as % of procedures. **C** MELD scores at the time of liver biopsy and after 72 h. **D** Distributions of grade I complications, multiple complications were possible in individual patients

Regarding post-procedural disease course, we did not observe deterioration of liver disease severity following liver biopsy in either group after 72 h or at the time of discharge (Fig. 1). Specifically, MELD scores remained stable after TB in patients with ACLF (ACLF-TB: 29.5 [24.25–32.25] vs. 29.5 [23.25–33.25]; *p* = 0.925) and without ACLF (LC-TB: 14 [10.5–15] vs. 13 [10–17.5]; *p* = 0.234). MELD scores in the ACLF-MB cohort showed a decrease (ACLF-MB: 27 [22–33] vs. 25 [21–33] *p* = 0.010) and remained stable in patients with cirrhosis (LC-MB: 16 [12–20] vs. 15.5 [10.75–20]; *n* = 46; *p* = 0.675).

Intraprocedural bleeding from the biopsy site was laparoscopically observed in nearly all (168/169; 99.4%) MB procedures, whereas no intraprocedural bleeding was (clinically/radiologically) noted during TB in either ACLF or LC patients (all *p* < 0.001). Major bleeding requiring instrument-based intervention was noted in 52/56 (92.9%) ACLF-MB and 94/113 (83.2%) of LC-MB procedures (*p* = 0.099). Further complications during LC-MB included a transient drop in oxygen saturation, without the need to interrupt the examination (1/113 = 1,8%), and a peritoneal bleeding, which spontaneously resolved (1/113 = 1.8%). In the LC-TB group, one case of liver capsule perforation was reported (1/32 = 3.1%).

Minor complications were frequent across all groups and occurred more often in ACLF-MB patients compared to LC-MB (82.1% vs. 53.1%, *p* < 0.001, Table 2), however, rates did not significantly differ between biopsy modalities within ACLF or LC patients. To address possible confounding by baseline disease severity and to test the robustness of our pre-specified complication definition, we performed two sensitivity analyses. First, applying a stricter complication definition that also classifies intraprocedural bleeding managed by argon plasma coagulation (APC) or compression hemostasis as a complication (rather than an expected part of the MB procedure, see Methods) raised the overall complication rate to 94.7% (54/57) in ACLF-MB and 87.7% (100/114) in LC-MB, without altering the relative comparison between groups. Second, within the ACLF subgroup, the difference in complication rates between MB (80.7%, 46/57) and TB (68.8%, 11/16) did not reach statistical significance (Fisher’s exact *p* = 0.32) and remained non-significant after adjusting for MELD score and platelet count (adjusted odds ratio for TB vs. MB 0.48, 95% CI 0.13–1.70, *p* = 0.253), indicating that the modality comparison is not materially confounded by the modest baseline severity imbalance, though statistical power remains limited by the small TB subgroup (*n* = 16). Grade I complications were common and predominantly mild and self-limiting across all groups. They occurred more frequently in ACLF-MB patients compared with LC-MB patients (73.2% vs. 52.2%, *p* = 0.012), while no significant differences were observed between biopsy modalities within the ACLF or LC groups. The distribution of specific grade I complications is illustrated in Fig. 1. A significant fraction of ACLF patients required transfusions within the first week after the procedure regardless of the biopsy modality. In contrast, transfusions after the procedure were rare in both LC groups. Transfusion rates were significantly higher in ACLF-MB patients than in LC-MB patients (21.4% vs. 8.8%, *p* = 0.024).

**Table 2:** Complications after liver biopsies

	ACLF-MB (*n* = 56)	ACLF-TB (*n* = 16)	*p*-Value	LC-MB (*n* = 113)	LC-TB (*n* = 32)	*p*-Value	ACLF-MB vs. LC-MB (*p*-Value)
Minor Complications(Clavien-Dindo I–II)	82.1% (46/56)	62.5% (10/16)	0.095	53.1% (60/113)	62.5% (20/32)	0.345	< 0.001
Major Complications (Clavien-Dindo III–V)	5.4% (3/56)	6.3% (1/16)	1.000	3.5% (4/113)	0%	0.576	0.686
Transfusions within 7 d of biopsy, n (%)	21.4% (12/56)	18.8% (3/16)	1.000	8.8% (10/113)	6.3% (2/32)	1.000	**0.022**
RBC — received, n (%)	21.4% (12/56)	37.5% (6/16)	0.205	7.1% (8/113)	6.3% (2/32)	1.000	**0.010**
Amount among recipients, median [Q1–Q3]	2.5 [2–6.25]	3 [1–5]	–	2.5 [2–3]	2.5 [2–3]*	–	–
Platelets — received, n (%)	12.5% (7/56)	25.0% (4/16)	0.247	2.7% (3/113)	0%	1.000	**0.016**
Amount among recipients, median [Q1–Q3]	3 [1–4]	1.5 [1–2.75]	–	1 [1–15]*	–	–	–
PPSB [IU] — received, n (%)	12.5% (7/56)	6.3% (1/16)	0.674	3.5% (4/113)	0%	0.576	**0.043**
Amount among recipients, median [Q1–Q3]	3500 [1200–10800]	9000†	–	1800 [1200–9300]	–	–	–
FFP — received, n (%)	8.9% (5/56‡)	12.5% (2/16)	0.647	5.4% (6/112‡)	0%	0.338	0.509
Amount among recipients, median [Q1–Q3]	10 [3–22]	9 [6–12]*	–	2 [2–13.75]	–	–	–
Fibrinogen [g] — received, n (%)	14.3% (8/56)	18.8% (3/16)	0.699	2.7% (3/113)	0%	1.000	**0.007**
Amount among recipients, median [Q1–Q3]	4 [2–13.75]	4 [1–4]*	–	6 [2–9]*	–	–	–
Vitamin K [mg] — received, n (%)	33.9% (19/56)	18.8% (3/16)	0.359	10.6% (12/113)	6.3% (2/32)	0.735	**< 0.001**
Amount among recipients, median [Q1–Q3]	30 [10–30]	10 [10–40]*	–	25 [12.5–40]	15 [10–20]*	–	–
Factor XIII [IU] — received, n (%)	0%	6.3% (1/16)	0.222	0.9% (1/113)	0%	1.000	1.000
Amount among recipients	–	1000†	–	750†	–	–	–
AT III [IU] — received, n (%)	0%	0%	–	0.9% (1/113)	0%	1.000	1.000
Amount among recipients	–	–	–	1000†	–	–	–
**ICU admission**	5.4% (3/56)	0%	1.000	3.5% (4/113)	0%	0.576	0.686
**Overall mortality**	12.3% (7/57)	25.0% (4/16)	1.000	0.9% (1/113)	0%	1.000	0.107
**Intervention-related mortality**	0%	0%	–	0%	0%	–	–

Values are n (%) unless noted. Transfusion receipt is shown as n (%), with administered volumes (amount > 0) summarized among recipients as median [IQR]. Between-group p-values for receipt reflect two-sided Fisher’s exact tests; volumes are reported descriptively given small sample sizes. *For *n* ≥ 4, values are median [range]. †Single case (*n* = 1). ‡Missing FFP data for one patient; receipt denominator uses available cases (ACLF-MB *n* = 56; LC-MB *n* = 112). Aborted biopsies are excluded from post-procedure complication and transfusion analyses

Major complications were rare and showed no statistically relevant differences between groups. No procedure-related deaths were observed. One grade III complication was observed in the LC-MB group, where secondary hemorrhage required surgical hemostasis. ICU admission due to postinterventional hemorrhage was infrequent and occurred only after MB (ACLF-MB: 5.4%, LC-MB: 3.5%, *p* = 0.687). In total, 11 of the 73 ACLF patients (15.1%) died within 6 weeks of the biopsy procedure (7/57 [12.3%] after ACLF-MB, 4/16 [25.0%] after ACLF-TB), reflecting the poor short-term prognosis of ACLF itself; a full patient-level chronology is provided in Supplementary Table ST5 In the ACLF-MB group, three patients died within 6 weeks of the procedure. Importantly, none of these cases were directly attributed to post-punctural bleeding: one patient with spontaneous bacterial peritonitis and prolonged kidney failure requiring terlipressin was found in cardiac arrest approximately 36 h after the procedure without evidence of intraabdominal bleeding assessed by ultrasound. One patient with advanced multiorgan failure (ACLFs > 65 points) developed fatal disseminated intravascular coagulation with diffuse hemorrhage and gastrointestinal bleeding and died 4 days after the procedure; an interim CT-scan had not revealed postpunctural intraabdominal hemorrhage. One patient developed recurring spontaneous bacterial peritonitis following postpunctural hemorrhage and died 39 days after the procedure. One patient in the ACLF-TB group died from septic shock but had already been admitted to the ICU prior to the biopsy procedure and therefore did not meet criteria for postinterventional ICU admission. The remaining seven deaths (four ACLF-MB, three ACLF-TB) reflected unambiguous, non-bleeding-associated progression of the underlying liver disease and/or infectious complications (e.g. hepatic encephalopathy with septic shock, progressive hepatocellular carcinoma, alcohol-associated steatohepatitis without treatment response, nosocomial pneumonia, and post-transplant infection); these cases were not individually adjudicated for a potential procedural contribution because no clinical suspicion of a bleeding- or procedure-related complication was raised at the time.

### Multivariate analysis

To identify independent predictors of relevant postinterventional bleeding, a multivariable logistic regression model was constructed with relevant bleeding as the binary outcome. The overall model was statistically significant (χ²(6) = 33.4, *p* < 0.001) and demonstrated good calibration in the Hosmer–Lemeshow test (*p* = 0.319). The model explained a moderate proportion of outcome variability (Nagelkerke R²=0.263) and correctly classified 87.5% of all cases. While the model correctly identified 97.9% of patients without relevant bleeding, it detected only 20.7% of patients who experienced bleeding events, indicating high specificity but poor sensitivity and thus limited utility for identifying patients at high risk of postinterventional bleeding. A higher MELD score was identified as the only independent predictor of relevant bleeding (OR = 1.14, 95% CI 1.05–1.24, *p* = 0.002). All other tested variables, including platelet count, presence of ACLF, biopsy type, cirrhosis etiology, and evidence of portal hypertension, showed no independent association with the outcome after adjustment (Table 3**).**

**Table 3:** Multivariate analysis of predictors of major postprocedural complications

Variable	Odds Ratio	95% CI (Lower - Upper)	*p*-value
MELD Score	1.141	1.052 − 1.239	**0.002**
Pre-interventional platelet count	0.997	0.991–1.004	0.420
Presence of ACLF	0.630	0.146–2.727	0.536
Type of biopsy (transjugular vs. mini-laparoscopy)	1.942	0.637–5.925	0.243
Cirrhosis etiology (non-alcoholic vs. alcoholic)	1.100	0.412–2.938	0.849
evidence of portal hypertension	1.111	0.279–4.419	0.881

ACLF = acute-on chronic liver failure

## Discussion

The present study sought to evaluate risks and diagnostic value of mini-laparoscopic versus transjugular biopsy in patients with ACLF, the most severe form of decompensated cirrhosis, where patient factors may associate with increased bleeding risk, while advanced tissue disintegration may impair tissue interpretability. Our retrospective analysis indicates that mini-laparoscopic liver biopsy is a safe modality for liver biopsy in the setting of ACLF, with a higher diagnostic yield compared to transjugular liver biopsy.

In terms of patient safety, the decision to proceed to liver biopsy requires careful clinical deliberation, particularly in the setting of ACLF. In fact, we only identified 73 patients who required urgent liver biopsy in the setting of ACLF in a 9-year observation period in our institution, a high-volume liver transplant center serving a population of ~ 5 million people. ACLF severity was mostly in the medium risk category as reflected by a median CLIFc ACLFs of ~ 50 points, and mini-laparoscopy is rarely performed in patients with circulatory, respiratory or advanced CNS failure in our center. However, our patient cohort exhibits disease severities comparable to benchmark ACLF studies [24, 25] and provides a general estimate for the role of liver biopsy when etiologic clarification and causative treatment may still substantially affect disease courses.

In our analysis, MB was generally preferred over TB, and TB was mostly performed either during TIPS placement, or when morbid obesity or a patient history of abdominal surgery precluded MB. Biopsy indications also differed modestly by clinical suspicion profile between groups (e.g., previously unknown/incidentally suspected cirrhosis prompted biopsy in 18.8% of ACLF-TB versus 5.3% of ACLF-MB patients; Supplementary Table ST1), consistent with a degree of clinical selection in modality choice that should be considered alongside the baseline disease-severity imbalances already discussed above. Percutaneous liver biopsy, by contrast, is usually avoided in patients with decompensated cirrhosis, particularly in the presence of ascites. This general concept is endorsed by our observation of very high rates of severe post-punctural bleeding requiring interventional hemostasis during mini-laparoscopy (83.2% in LC, 92.9% in ACLF), where bleeding is directly visible. Bleeding was generally well-controlled by different coagulation techniques and rarely translated into clinically relevant hemorrhage. Pre-interventional administration of blood products and coagulation factors was more prevalent in ACLF compared to LC patients for several products. Differences between biopsy modalities within the respective ACLF and LC cohorts were less pronounced. While this indicates that coagulation abnormalities and suspected bleeding risk were generally more severe in ACLF, they did not inform biopsy modality and reflect the clinical practice of pre-emptive optimization of coagulation parameters prior to invasive procedures, which had been broadly implemented during the study period but is no longer part of current clinical practice guidelines [6, 8].

We observed a robust trend towards higher rates of minor periprocedural complications after MB compared to TB, particularly with regards to bleeding at the cutaneous biopsy sites and post-procedural hypotension, but somewhat lower rates of nausea/vomiting and post-procedural pain, which is relevant for patient counselling, but in general of subordinate clinical relevance. Post-interventional transfusion requirements were more frequent in ACLF than in stable cirrhosis, consistent with the greater clinical severity and coagulation abnormalities in ACLF. Importantly, major complications following liver biopsy were comparable between both methods. Overall, 11 of 73 ACLF patients (15.1%) died within 6 weeks of liver biopsy (7/57 [12.3%] after ACLF-MB, 4/16 [25.0%] after ACLF-TB, consistent with the generally poor short-term prognosis of ACLF. Among these, three ACLF-MB patients died within 6 weeks after mini-laparoscopic liver biopsy, two of whom had ACLF grade III with dismal short-term prognosis even without any intervention based on current prognostication models, and all patients had or developed severe infections as well-known precipitators of further clinical demise [26]. While overt post-punctural bleeding was ruled out as a direct cause of death in these cases, even minor physiologic disturbances including sedation, laparoscopy and minor post-punctural bleeding as well as pre- or postinterventional transfusion of blood products may have contributed to clinical deterioration in these particular cases, and supreme attention should be paid to the indication of liver biopsy, careful periprocedural patient management and post-interventional surveillance.

In general, however, we did not observe a notable increase in patient disease severity following liver biopsy, as reflected by comparable MELD scores before and 72 h after liver biopsy. Importantly, biopsy modality itself was not independently associated with relevant bleeding, and our multivariate analysis identified MELD score as the only independent predictor of relevant hemorrhage. Notably, back-calculating the effective number of bleeding events from the model’s published performance characteristics (sensitivity 20.7%, specificity 97.9%, accuracy 87.5%, χ²(6) = 33.4, *N* = 217) yields an estimated events-per-variable (EPV) ratio of approximately 4.8 for this six-predictor model, below the conventional EPV ≥ 10 threshold recommended for stable multivariable logistic regression estimates. Odds ratios for the weaker predictors in this model should therefore be interpreted with appropriate caution, and external validation in larger, multi-center cohorts is warranted. The limited sensitivity of the model, however, underscores the well-established notion that bleeding risk in this population cannot be reliably predicted based on routine clinical parameters alone, and individual patient factors (particularly infections) may crucially increase risk even for very severe complications. Taken together, the MB procedure is generally safe and postprocedural complications are usually mild even in the presence of ACLF, but inherent limitations of risk stratification in ACLF necessitate procedural strategies that enable immediate bleeding control and management of complications. Of note, TB was not associated with any intraprocedural bleeding, consistent with previous studies that showed its safety in patients with coagulopathy [27].

In terms of diagnostic yield, MB was associated with a higher diagnostic yield than TB, mainly attributable to the smaller core-length as well as the higher fragmentation rate of TB compared to MB samples. This observation is consistent with a previous analysis suggesting significantly higher biopsy quality of MB compared to TB samples [17]. Our findings translate this finding into the area of cirrhosis/ACLF, where heterogeneous distribution of cell death, inflammation and cholestasis as well as the presence of advanced severe fibrosis promote tissue disintegration and increase the likelihood of insufficient tissue quality [3, 18]. Likewise, in terms of diagnostic value, MB provided a higher diagnostic yield than TB without an increase in major complication rates. Consequently, the transjugular approach resulted in inconclusive specimens in 37.6% of patients with ACLF (31.3% technical + 6.3% diagnostic failure), and 43.8% (37.5% technical + 6.3% diagnostic failure) in patients with stable cirrhosis. These rates are significantly inferior compared to non-conclusive specimens in 3.6% of ACLF patients and 17.7% in stable cirrhosis patients biopsied via laparoscopy - all of which were diagnostic failures with only two technical failures, in which large volume ascites and peritoneal adhesions precluded liver biopsy. A detailed breakdown of the final underlying liver disease diagnoses reached after biopsy, established via histology (MB) or a composite clinical/laboratory/imaging work-up (TB and non-invasive LC assessment), is provided in Supplementary Table ST6; alcohol-associated and autoimmune cirrhosis were the most common diagnoses in both groups, while a cryptogenic (i.e., etiologically unresolved) diagnosis was ultimately reached in 8.8% of ACLF-MB, 31.3% of ACLF-TB, 16.7% of LC-MB and 37.5% of LC-TB patients (18.7% of the total cohort, *n* = 219). In a complementary sensitivity analysis restricted specifically to the subset of patients in whom biopsy was indicated because the underlying etiology was unclear at baseline (Supplementary Table ST2), MB again established a diagnosis significantly more often than TB in the ACLF cohort (28/32 [87.5%] vs. 4/8 [50.0%]; *p* = 0.037), with a concordant, non-significant trend in the LC cohort (49/69 [71.0%] vs. 14/27 [51.9%]; *p* = 0.076) — corroborating the overall etiology-determination rates already reported in the Results (ACLF: 5.3% vs. 37.5% undetermined, *p* = 0.003; LC: 18.4% vs. 43.8% undetermined, *p* = 0.005). As reported in the Results, the elevated rate of undetermined etiology after MB in stable cirrhosis (LC-MB) was not attributable to technical/sampling failure, since all LC-MB specimens (113/113) were technically adequate for evaluation; rather, this diagnostic failure specifically reflected extensive fibrosis or inflammatory changes obscuring the underlying histological pattern. We hypothesize that this relates to the generally more advanced, “burnt-out” fibrotic architecture of stable end-stage cirrhosis, in which extensive bridging fibrosis and regenerative nodules can obscure the original etiologic pattern despite adequate sample quality; ACLF specimens, by contrast, more often display acute or subacute pathological changes (e.g. hepatocyte ballooning, cholestasis, steatosis, inflammatory infiltrates) that remain etiologically informative even against a cirrhotic background. This interpretation is offered as a hypothesis warranting further histopathological investigation, not an established mechanism.

Formulating causal inferences from this retrospective analysis is naturally impeded by its retrospective nature. As such, the biopsy modality was determined by clinical assessment and did not follow a pre-specified decision tree. We do, however, provide an overview of risks and benefits of different biopsy modalities in a considerably large cohort of critically ill patients, where randomization is difficult to achieve and clinical deterioration and complications can be inferred by both the underlying disease as well as the procedure itself. A further inherent limitation is that MB and TB samples were also preferentially obtained from different liver lobes (MB: left lobe in 53/80 [66%] ACLF and 108/181 [60%] LC biopsies; TB: right lobe in 20/24 [83%] ACLF and 28/34 [82%] LC biopsies), introducing anatomical sampling location as an additional confound that cannot be fully disentangled from biopsy modality itself in this retrospective comparison. Based on our overall findings, we propose that MB be considered a first-line biopsy modality whenever technically feasible in ACLF and LC patients requiring histological clarification, given its higher diagnostic yield and comparable major-complication risk; TB should be preferentially reserved for patients with severe obesity precluding safe abdominal access, extensive abdominal adhesions from prior surgery, a concurrent indication for TIPS placement, or therapeutic anticoagulation that cannot be safely interrupted. This pragmatic algorithm reflects our institutional practice during the study period and requires prospective validation.

## Supplementary Information

Below is the link to the electronic supplementary material.

Supplementary Material 1

Supplementary Material 2

Supplementary Material 3

Supplementary Material 4

Supplementary Material 5

Supplementary Material 6

## Data Availability

The datasets used and/or analysed during the current study are available from the corresponding author on reasonable request.

## Abbreviations

ACLF: acute-on chronic liver failure
APC: argon plasma coagulation
AT: antithrombin
FFP: fresh frozen plasma
ICU: intensive care unit
IU: International Units
LC: liver cirrhosis
LT: liver transplantation
MASLD: metabolic dysfunction-associated steatotic liver disease
MB: mini-laparoscopic liver biopsy
RBC: red blood cells
TB: transjugular liver biopsy
PPSB: prothrombin complex concentrate
