# Supplementary material for: Superior diagnostic yield of mini-laparoscopy over transjugular liver biopsy in acute-on-chronic liver failure: a case-control study
Source: BMC Gastroenterol. 2026 Oct 5;26:616. doi: 10.1186/s12876-026-05401-4 (PMC13640294; doi:10.1186/s12876-026-05401-4)
Supplement: Supplementary file 1 [file 12876_2026_5401_MOESM1_ESM.docx]

| **Indication for biopsy** | **ACLF-MB (n=57)** | **ACLF-TB (n=16)** | **LC-MB (n=114)** | **LC-TB (n=32)** |
| --- | --- | --- | --- | --- |
| Unknown underlying etiology | 33 (57.9%) | 8 (50.0%) | 69 (60.5%) | 27 (84.4%) |
| Previously unknown/suspected cirrhosis | 3 (5.3%) | 3 (18.8%) | 38 (33.3%) | 7 (21.9%) |
| Suspected alcohol-related liver disease | 16 (28.1%) | 5 (31.3%) | 11 (9.6%) | – |
| Suspected malignancy | 6 (10.5%) | 1 (6.3%) | 12 (10.5%) | 1 (3.1%) |
| Suspected autoimmune disease† | 3 (5.3%) | 1 (6.3%) | 14 (12.3%) | 3 (9.4%) |
| Suspected viral hepatitis | – | 2 (12.5%) | 11 (9.6%) | – |
| Suspected metabolic/infiltrative disease‡ | – | – | 1 (0.9%) | 1 (3.1%) |
| Suspected drug-induced liver injury | – | – | 1 (0.9%) | – |

Supplementary Table ST1. Indications for liver biopsy, by group and biopsy modality. †Autoimmune hepatitis, primary biliary cholangitis, primary sclerosing cholangitis, or overlap syndrome. ‡MASLD or amyloidosis. Patients may have more than one listed indication; percentages are column percentages of the group total and may not sum to 100%.
