# Supplementary material for: Superior diagnostic yield of mini-laparoscopy over transjugular liver biopsy in acute-on-chronic liver failure: a case-control study
Source: BMC Gastroenterol. 2026 Oct 5;26:616. doi: 10.1186/s12876-026-05401-4 (PMC13640294; doi:10.1186/s12876-026-05401-4)
Supplement: Supplementary file 2 [file 12876_2026_5401_MOESM2_ESM.docx]

| **Diagnostic question (indication-restricted subset)** | **MB** | **TB** | **p-value** |
| --- | --- | --- | --- |
| Cirrhosis diagnosis, ACLF (previously unknown/suspected cirrhosis subset) | 3/3 (100%) | 3/3 (100%) | 1.000 |
| Cirrhosis diagnosis, LC (previously unknown/suspected cirrhosis subset) | 34/38 (89.5%) | 5/7 (71.4%) | 0.230 |
| Etiology diagnosis, ACLF (previously unclear etiology subset) | 28/32 (87.5%) | 4/8 (50.0%) | 0.037 |
| Etiology diagnosis, LC (previously unclear etiology subset) | 49/69 (71.0%) | 14/27 (51.9%) | 0.076 |

Supplementary Table ST2: Diagnostic influence of biopsy in patients with a previously unresolved clinical question. Denominators restrict to patients whose biopsy indication was specifically "unknown underlying etiology" (etiology rows) or "previously unknown/suspected cirrhosis" (cirrhosis rows), i.e., patients for whom the diagnostic question was genuinely open before biopsy. p-values from Fisher’s exact test.
