# Supplementary material for: Superior diagnostic yield of mini-laparoscopy over transjugular liver biopsy in acute-on-chronic liver failure: a case-control study
Source: BMC Gastroenterol. 2026 Oct 5;26:616. doi: 10.1186/s12876-026-05401-4 (PMC13640294; doi:10.1186/s12876-026-05401-4)
Supplement: Supplementary file 3 [file 12876_2026_5401_MOESM3_ESM.docx]

| **Therapeutic consequence** | **MB** | **TB** | **p-value** |
| --- | --- | --- | --- |
| LT evaluation/planning (ACLF) | 10/56 (17.9%) | 1/16 (6.3%) | 0.436 |
| LT evaluation/planning (LC) | 13/114 (11.4%) | 1/32 (3.1%) | 0.305 |
| Treatment discontinued/adjusted (ACLF) | 5/56 (8.9%) | 2/16 (12.5%) | 0.647 |
| Treatment discontinued/adjusted (LC) | 6/114 (5.3%) | 0/32 (0.0%) | 0.339 |
| Malignancy excluded/detected (ACLF) | 3/56 (5.4%) | 0/16 (0.0%) | 1.000 |
| Malignancy excluded/detected (LC) | 6/114 (5.3%) | 0/32 (0.0%) | 0.339 |

Supplementary Table ST3. Therapeutic consequences of liver biopsy, by group and biopsy modality LT = liver transplantation. p-values from Fisher’s exact test. None of the modality comparisons reached statistical significance, though numerical differences favor MB for LT evaluation/planning and for treatment discontinuation/adjustment in both groups.
