# Supplementary material for: Superior diagnostic yield of mini-laparoscopy over transjugular liver biopsy in acute-on-chronic liver failure: a case-control study
Source: BMC Gastroenterol. 2026 Oct 5;26:616. doi: 10.1186/s12876-026-05401-4 (PMC13640294; doi:10.1186/s12876-026-05401-4)
Supplement: Supplementary file 4 [file 12876_2026_5401_MOESM4_ESM.docx]

| Transfusion product | ACLF-MB (n=57) | ACLF-TB (n=16) | p | LC-MB (n=114) | LC-TB (n=32) | p | P (ACLF-MB vs. LC-MB) |
| --- | --- | --- | --- | --- | --- | --- | --- |
| **Red blood cells (RBC), units** | | | | | | | |
| Received, n (%) | 6 (10.5%) | 5 (31.3%) | .055 | 2 (1.8%) | 4 (12.5%) | **.021** | **.017** |
| Amount among recipients, median [IQR] | 2 [1.75–5.25] | 2 [1–3.5] | – | 1 [1–1] | 2 [1.25–2] | – | – |
| **Fibrinogen, g** | | | | | | | |
| Received, n (%) | 9 (15.8%) | 2 (12.5%) | 1.000 | 3 (2.6%) | 1 (3.1%) | 1.000 | **.003** |
| Amount among recipients, median [IQR] | 2 [2–4] | 5.5 (n=2) | – | 2 [2–2] | n=1 | – | – |
| **PCC, IU** | | | | | | | |
| Received, n (%) | 10 (17.5%) | 2 (12.5%) | 1.000 | 1/113 (0.9%) | 0/32 (0%) | 1.000 | **<.001** |
| Amount among recipients, median [IQR] | 1800 [1050–2400] | 3150 (n=2) | – | n=1 | – | – | – |
| **FFP, units** | | | | | | | |
| Received, n (%) | 6 (10.5%) | 2 (12.5%) | 1.000 | 5 (4.4%) | 1 (3.1%) | 1.000 | .183 |
| Amount among recipients, median [IQR] | 4 [2.75–4.5] | 4 [4–4] | – | 2 [1.5–6.5] | n=1 | – | – |
| **Vitamin K, mg** | | | | | | | |
| Received, n (%) | 24 (42.1%) | 4 (25.0%) | .257 | 14 (12.3%) | 3 (9.4%) | .765 | **<.001** |
| Amount among recipients, median [IQR] | 20 [12.5–30] | 35 [22.5–40] | – | 20 [17.5–30] | 10 [10–10] | – | – |
| **Platelets, units** | | | | | | | |
| Received, n (%) | 8 (14.0%) | 4 (25.0%) | .444 | 6 (5.3%) | 6 (18.8%) | .**024** | .073 |
| Amount among recipients, median [IQR] | 1 [1–1] | 1 [1–2.5] | – | 1 [1–1.25] | 1 [1–2] | – | – |
| **Factor XIII, IU** | | | | | | | |
| Received, n (%) | 0 (0%) | 1 (6.3%) | .219 | 0 (0%) | 0 (0%) | – | – |
| Amount among recipients, median [IQR] | – | n=1 | – | – | – | – | – |
| **Antithrombin III, IU** | | | | | | | |
| Received, n (%) | 0 (0%) | 1 (6.3%) | .219 | 0/113 (0%) | 0/32 (0%) | – | – |
| Amount among recipients, median [IQR] | – | n=1 | – | – | – | – | – |

Supplementary Table ST4: Pre-interventional use of blood products. Received values are n (%). Between-group p values for receipt of each product are two-sided Fisher’s exact tests. Amounts are shown descriptively among patients who received the respective product (>0), as median [25th–75th percentile]. No p values are reported for recipient-only amounts because recipient numbers were very small for several products. For n=1 or n=2, an IQR was not reported when it could not be meaningfully estimated; “–” indicates not applicable/not estimable. PCC, prothrombin complex concentrate; FFP, fresh frozen plasma; IU, international units; MB, mini-laparoscopic biopsy; TB, transjugular biopsy. For PPSB and antithrombin III, LC-MB denominators are 113 because of one missing value. Statistically significant p values (p < 0.05) are shown in bold.
