# Supplementary material for: Superior diagnostic yield of mini-laparoscopy over transjugular liver biopsy in acute-on-chronic liver failure: a case-control study
Source: BMC Gastroenterol. 2026 Oct 5;26:616. doi: 10.1186/s12876-026-05401-4 (PMC13640294; doi:10.1186/s12876-026-05401-4)
Supplement: Supplementary file 5 [file 12876_2026_5401_MOESM5_ESM.docx]

| **ID*** | **Day of death** | **Cause of death (summary)** | **Bleeding phenomenon?** | **Puncture-site bleeding assessed** |
| --- | --- | --- | --- | --- |
| **MB1** | 2 | Suspected variceal/hemorrhagic shock; cardiac arrest ~36 h post-biopsy | Yes (non-puncture site) | Yes — ultrasound showed no bleeding |
| **MB2** | 2 | Decompensated cirrhosis with hepatic encephalopathy grade III–IV, septic shock/multiorgan failure, AKI/HRS | No | N/A |
| **MB3** | 3 | Progressive liver failure with metastatic hepatocellular carcinoma and infection | No | N/A |
| **MB4** | 4 | Disseminated intravascular coagulation with diffuse hemorrhage/hematochezia | Yes (non-puncture site) | Yes — CT excluded post-punctural bleeding |
| **MB5** | 7 | Respiratory failure; not a liver-transplant candidate; palliative care | No | N/A |
| **MB6** | 10 | Progressive alcohol-associated steatohepatitis; minor subcapsular hematoma/blood-tinged ascites, conservatively managed | Yes (minor, managed conservatively) | Death attributed to ASH/infection, not to this bleeding |
| **MB7** | 39 | Recurrent spontaneous bacterial peritonitis leading to refractory septic shock | No | N/A |
| **TB1** | 4 | Pre-existing sepsis at time of biopsy; incidental hemochromatosis diagnosis | No | N/A |
| **TB2** | 4 | Hematemesis from bleeding ligature ulcers at a variceal band site | Yes (non-puncture site) | Yes — CT excluded puncture-site bleeding |
| **TB3** | 12 | Nosocomial pneumonia with HRS-related AKI; care not escalated | No | N/A |
| **TB4** | 29 | Aspergillus pneumonia/sepsis after liver transplantation | No | N/A |

Supplementary Table ST5: Chronology of all 11 deaths within 6 weeks of liver biopsy, anonymized labels N/A = not applicable (no bleeding phenomenon was observed or reported).
