# Supplementary material for: Superior diagnostic yield of mini-laparoscopy over transjugular liver biopsy in acute-on-chronic liver failure: a case-control study
Source: BMC Gastroenterol. 2026 Oct 5;26:616. doi: 10.1186/s12876-026-05401-4 (PMC13640294; doi:10.1186/s12876-026-05401-4)
Supplement: Supplementary file 6 [file 12876_2026_5401_MOESM6_ESM.docx]

| **Final diagnosis** | **ACLF-MB (n=57)** | **ACLF-TB (n=16)** | **LC-MB (n=114)** | **LC-TB (n=32)** | **Total (n=219)** |
| --- | --- | --- | --- | --- | --- |
| Alcohol-related liver disease | 35 (61.4%) | 7 (43.8%) | 38 (33.3%) | 11 (34.4%) | 91 (41.6%) |
| Cryptogenic cirrhosis | 5 (8.8%) | 5 (31.3%) | 19 (16.7%) | 12 (37.5%) | 41 (18.7%) |
| Autoimmune liver disease | 6 (10.5%) | 1 (6.3%) | 27 (23.7%) | 3 (9.4%) | 37 (16.9%) |
| Viral hepatitis | 6 (10.5%) | 1 (6.3%) | 17 (14.9%) | – | 24 (11.0%) |
| Metabolic / infiltrative* | 3 (5.3%) | 1 (6.3%) | 8 (7.0%) | 5 (15.6%) | 17 (7.8%) |
| Other | – | – | 2 (1.8%) | 1 (3.1%) | 3 (1.4%) |
| Secondary sclerosing cholangitis | – | 1 (6.3%) | 1 (0.9%) | – | 2 (0.9%) |
| Toxic / drug-induced | – | – | 2 (1.8%) | – | 2 (0.9%) |
| Alcohol-related + viral | 2 (3.5%) | – | – | – | 2 (0.9%) |

Supplementary Table ST6: Final diagnosis after liver biopsy, by group and biopsy modality
